# The Physical Economy of the United States of America

**DOI:** 10.1111/j.1530-9290.2011.00404.x

**Published:** 2011-12-01

**Authors:** Sylvia Gierlinger, Fridolin Krausmann

**Keywords:** dematerialization, industrial ecology, material flow accounting, material flow analysis (MFA), resource productivity, societal metabolism

## Abstract

The United States is not only the world's largest economy, but it is also one of the world's largest consumers of natural resources. The country, which is inhabited by some 5% of the world's population, uses roughly one-fifth of the global primary energy supply and 15% of all extracted materials. This article explores long-term trends and patterns of material use in the United States. Based on a material flow account (MFA) that is fully consistent with current standards of economy-wide MFAs and covers domestic extraction, imports, and exports of materials for a 135-year period, we investigated the evolution of the U.S. industrial metabolism. This process was characterized by an 18-fold increase in material consumption, a multiplication of material use per capita, and a shift from renewable biomass toward mineral and fossil resources. In spite of considerable improvements in material intensity, no dematerialization has happened so far; in contrast to other high-income countries, material use has not stabilized since the 1970s, but has continued to grow. This article compares patterns and trends of material use in the United States with those in Japan and the United Kingdom and discusses the factors underlying the disproportionately high level of U.S. per capita resource consumption.

## Introduction

In the late-nineteenth century the United States challenged the United Kingdom as the leading economic power, and it has since occupied a predominant role in the world economy; at the turn of the twenty-first century, the United States produced almost 30% of global gross domestic product (GDP). The United States is not only the world's largest economy, it is also the world's largest consumer of natural resources. In the year 2005, the country, which contained merely 5% of the global population, used roughly one-fifth of the global primary energy supply and 15% of all extracted materials. By output, the United States emitted 21% of global carbon dioxide (CO_2_) emissions from fossil fuels and cement production (Marland et al. 2007). This article explores the long-term historical development of the physical economy of the United States and the evolution of its industrial metabolism. It presents a material flow account (MFA) for the period 1870–2005 and investigates the extraction, trade, and consumption of materials from main material groups.

Several previous studies have explored historical trends of energy and material use in the United States. Schurr and Netschert (1960) presented the first analysis of the development of U.S. (technical) primary energy use since the mid-nineteenth century. More recently, Ayres and colleagues (2003) investigated primary energy inputs and useful work supply (exergy use) for the United States for the period 1900–1998 (see also Warr et al. 2010). Material inputs into the U.S. economy have also been studied: An initial database compiled by Matos and Wagner (1998) focused on raw materials for industrial use and presented yearly data for the period 1900–1995. It has since been updated to include 2006 as the most recent year (see Rogich and Matos 2002; Rogich et al. 2008; Matos 2009). Although covering important parts of social metabolism, these data are not fully consistent with current MFA system boundaries and accounting principles: Important materials like the largest component of biomass flows and most fossil energy carriers are not considered; others, such as recycled materials, are accounted for as inputs, in deviation from MFA standards. Ayres and colleagues (2004) used their database on long-term exergy and material use to analyze whether or not the U.S. economy was dematerializing in the twentieth century. These datasets focus on apparent consumption and do not distinguish between extraction, imports, and exports. Finally, the United States was one case in the seminal comparative MFA study initiated by the World Resources Institute (Adriaanse et al. 1997). From this study, detailed MFA data exist, but only for the period 1975–1993; this dataset was later updated to the year 2000 (Rogich et al. 2008).

We compiled a long-term historical database for material flows in the U.S. economy that is fully consistent with current standards of economy-wide MFA. Compared with previous studies, our dataset considerably expands the observed time period and the flows and materials covered. It provides detailed data for domestic extraction, imports, and exports in annual resolution for a 135-year period and distinguishes up to 60 material groups. MFA data and the derived indicators, such as domestic material consumption or physical trade balance, are comparable to other long-term MFA studies (Schandl and Schulz 2002; Krausmann et al. 2009, 2011) and are publicly available from http://www.uni-klu.ac.at/socec/inhalt/1088.htm.

This article explores the metabolic transition in the United States and the emergence of its industrial metabolism, a process characterized by an overall growth in material use far greater than the pace of population growth, and by fundamental changes in the composition of material use. We analyze how material input and use have changed and are changing in relation to economic development, population growth, and energy consumption. Furthermore, we compare U.S. material throughput to that of other countries and discuss the significance of the U.S. economy for global material use.

After a brief description of methods and data sources in the following section, the article presents time series data on extraction, trade, and material consumption for the period 1870–2005 (“Results”). The “Socio-metabolic transitions” section discusses different phases of the metabolic transition in the United States in relation to economic and political developments. Following that we analyze the relationship between material flows and economic development, and finally, we discuss U.S. resource use patterns in a global context.

## Methods and Data

For compiling a comprehensive database on the long-term development of material and energy flows in the U.S. economy,^1^ standard methods of economy-wide material flow accounting were applied (Eurostat 2009). This study quantified used extraction, imports, and exports of materials and used data from a range of national and international statistical sources. Estimation procedures were applied for materials that were not reported in statistical sources. The MFA database follows the materials classification used by Eurostat (2009) and distinguishes between 50 and 60 material groups for (used) domestic extraction, imports, and exports. Unused extraction or indirect flows (upstream material flows of traded products) were not considered. In this article, data and indicators are presented for four main material groups: biomass, fossil energy carriers, ores, and nonmetallic minerals. Furthermore, a distinction between renewable (biomass) and nonrenewable (mineral and fossil) materials is made.

We calculated the following material flow indicators (see Fischer-Kowalski et al. 2011): domestic extraction (DE); imports and exports; domestic material consumption (DMC), which is defined as DE plus imports minus exports; physical trade balance (PTB), which is defined as imports minus exports; and material intensity (MI), which is defined as DMC per unit of GDP and is the inverse of material productivity. We also refer to per capita values of domestic material and energy consumption as metabolic rates.

### Data sources

We based our long-term historical reconstruction of material flows in the United States on three major sources. First, we used the Historical Statistics of the United States (HSUS; U.S. Bureau of the Census 1975),^2^ which provides a comprehensive collection of statistical time series data covering a wide range of socioeconomic variables, including resource extraction from colonial times to present. Second, in the 1960s and 1970s Resources for the Future^3^ commissioned a number of studies on U.S. historical resource trends. These yielded two comprehensive data compilations—Potter and Christy (1962) and Manthy (1978)—that provide annual data on production of and trade for a large number of raw materials and commodities since the late-nineteenth century. Third, the U.S. Geological Survey (USGS) maintains a material flow database and provides time series data on most mineral materials from 1900 to recent years (Kelly and Matos 2008). In addition to these national sources, we used a range of international databases that provide data for more recent decades. As a general rule, international data were cross-checked with national sources for selected overlapping years in order to ensure consistency between sources and to avoid statistical breaks. Cross-checks have shown that while the pre-1960 sources generally cover a smaller number of materials, the difference in overall mass flows covered is negligible.

Crop harvest was fully covered in national and international statistical sources. We used Potter and Christy (1962), the HSUS (1975), Manthy (1978), and FAO (2005, 2009) to compile data on crops.

The extraction of used crop residues was estimated on the basis of time-dependent harvest factors and recovery rates for major crops. These were based on information derived from Wirsenius (2000) and Cunfer and Krausmann (2009). Grazed biomass was estimated on the basis of a simplified feed balance (Krausmann et al. 2008a). Data on livestock, livestock production, and feed availability were taken from the HSUS (1975) and FAO (2009). Feed demand (kilograms [kg]^4^ dry matter per head per day) for cattle was calculated on the basis of ruminant production (milk, meat) and assumptions on changes in livestock productivity were derived from Krausmann and colleagues (2008a) and Cunfer and Krausmann (2009). Feed demand factors for all other grazers were kept constant over time. In order to determine the amount of grazed biomass, available fodder crops, market feed, and crop residues were subtracted from the calculated total dry matter feed demand. For early years, no information on market feed was available. Based on information available for the 1960s (FAO 2009), we assumed a market feed share of 15% of total dry matter feed demand. Data on fish catch (excluding aquaculture, which is considered an internal transfer in MFA) were taken from the HSUS (1975), Manthy (1978), and FAO (2006).

Data on the extraction of industrial roundwood were derived from the HSUS for years prior to 1960 and from FAO 2009 for the period 1961–2005. For the years 1870–1900, the HSUS reports lumber and pulpwood only. The inclusion of other wood items in statistical reporting after 1900 results in a statistical break; therefore, numbers for industrial roundwood production increase by 25% after 1899. Fuel wood data are available from energy statistics (Schurr and Netschert 1960; IEA 2007) and production statistics (Manthy 1978; Howard 2007). While both sources show a very similar development of fuel wood use over time, the values derived from energy statistics are up to 40% higher than those from production statistic. To avoid double counting due to reuse of wood, we followed a conservative approach and used the lower values from the production statistics.

Data on the extraction of fossil energy carriers are well covered in statistical sources. For the period 1870–1960, data on the extraction of coal, oil, and natural gas were taken from the HSUS (1975). For the more recent years (1961–2005), data are from IEA (2007). Data on peat extraction were taken from Kelly and Matos (2008).

Data on the extraction of ores are very well documented by the USGS (Kelly and Matos 2008) from 1900 on. Data for the years prior to 1900 are from the HSUS and were complemented with data from Manthy (1978). Only for iron and bauxite do statistical sources report production in terms of gross ore. All other metals are reported in terms of metal content. To arrive at gross ore production, as required in MFA, we used information on coupled production and metal content, derived from the USGS (e.g., U.S. Bureau of Mines 1987). Gross ore values were calculated only for the main metals mined through coupled production to avoid double counting. For copper, which is the largest mass flow of non-iron ores, we assumed that ore grades declined from 2.5% in 1880 to 0.5% in 1975 based on ore grades given in Ayres and colleagues (2004). For all other metals, current ore grades were used, which results in an overestimation of gross ores in earlier periods when grades of some domestic ores were higher (cf. Mudd 2009; West 2011).

We used data on the extraction of nonmetallic minerals from the HSUS (1975) and Kelly and Matos (2008). Data on the extraction of natural aggregates (sand, gravel, crushed stone) reported in the HSUS are not consistent with the much lower numbers provided in Kelly and Matos (2008). Therefore we estimated the demand for sand, gravel, and crushed stone based on the production and use of cement, concrete, and asphalt (see Eurostat 2009; Krausmann et al. 2009; Schandl and West 2010). We applied standard coefficients on the ratio of sand and gravel to cement and bitumen in concrete and asphalt in order to extrapolate natural aggregates use. Data on the production and consumption of cement and bitumen were taken from Abraham (1945), the HSUS (1975), IEA (2007), and Kelly and Matos (2008). The results of this estimate match well with data reported in the HSUS (1975) and those estimated by Matos (2009); trends over time are similar in both estimates (see supporting information available on the Journal's Web site for details).

Comprehensive trade data are difficult to obtain. Most sources only cover trade with raw materials and semimanufactured products, but mass flows of manufactured products are rare. Data on the trade of biomass, fossil energy carriers, and products thereof were derived from Potter and Christy (1962) and Manthy (1978) for the years 1870–1960. From 1961 on we used FAO (2009) data for trade of agricultural and forestry products and IEA (2007) data for trade of fossil energy carriers and petrochemical products. For wood, data on net trade were only available for the years 1870–1950. Trade data on nonmetallic minerals and ores and semimanufactured metal products were taken from Potter and Christy (1962) for the period 1870–1899 and from Kelly and Matos (2008) for the period 1900–2005.

We used data from the United Nations (UN) Comtrade database (United Nations Statistical Division 2008) to cross-check our results and to quantify underestimations due to incomplete coverage of trade of manufactured products like furniture, textiles, machinery, and vehicles. Comtrade data are available from 1962 to the present, but for most years data on physical trade flows are fragmentary. Comprehensive data on imports and exports are available only for the years 1978, 1985–1988, and 2005. An analysis of Comtrade data for the years 1978 and 2005 revealed that manufactured products not considered in our accounting amount to 10% to 20% of total imports and 4% to 11% of exports (see the Supporting Information on the Web for details). In terms of net trade (physical imports minus physical exports), the underestimation is much smaller. Underestimation is highest for imports and exports of metal products (vehicles, machinery), petrochemical products (organic chemicals, plastics) and so-called other products, which are not assigned to a specific material group.

Data on material flows were used to calculate the total primary energy supply (TPES) for the entire time period. We converted flows of fuel wood and fossil energy carriers into energy units using material-specific gross calorific values. Energy flow data derived from MFA were supplemented with energy inputs from hydropower, nuclear heat, and geothermal sources. Data on electricity output from hydro and nuclear power plants (U.S. Bureau of the Census 1975 and IEA 2007) were converted into primary energy input by applying coefficients derived from information on average conversion efficiencies (Warr et al. 2010) (see the supporting information on the Web for details).

## Results

**Figure 1:**
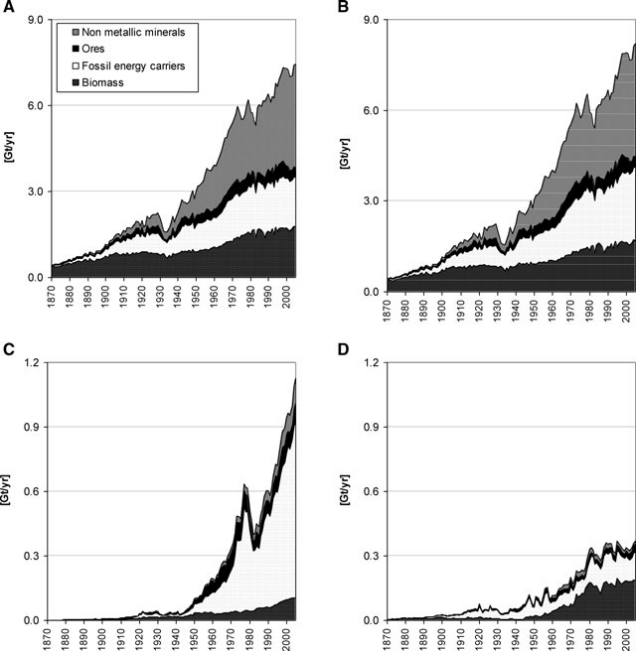
Material flows in the United States in gigatons/year (Gt/yr), 1870–2005: (a) domestic extraction of raw materials, (b) domestic material consumption, (c) imports, and (d) exports of raw materials and semimanufactured products. The segments in the graphs follow a bottom to top order of biomass, fossil energy carriers, ores, non metallic minerals.

Figure 1 shows the development of U.S. DE, DMC, and physical imports and exports from 1870–2005 by four main material groups.^5^

DE of materials rose from 0.4 gigatons (Gt)^6^ in 1870 to 7.4 Gt in 2005, corresponding to a 17-fold increase. Extraction grew throughout almost the entire period; decline during consecutive years was a rare exception. The most significant slumps in DE occurred during the Great Depression in the early 1930s and in the aftermath of oil price spikes in 1973 and 1979. Essentially all periods of recession (e.g., in 1990 and in 2000) were mirrored by declining rates of resource extraction. At the beginning of the observed period, DE was dominated by biomass. The fraction of biomass declined rapidly, from 86% in 1870 to below 50% in 1911 to 25% in the early 1960s, where it has remained. Even though the relative contribution of biomass to DE declined continuously, the total mass flow multiplied: DE of biomass grew fivefold from 0.39 gigatons per year (Gt/yr) in 1870 to 1.76 Gt/yr in 2005. Since the beginning of the twentieth century, mineral and fossil materials have come to dominate domestic extraction; their share has increased from 14% of DE to roughly three-quarters of total DE. In 2005, non-metallic minerals, mostly natural aggregates, accounted for 3.6 Gt, or almost 50% of DE, and fossil energy carriers accounted for 1.7 Gt. Extraction of metal ores grew at the same rate as DE of nonmetallic minerals until the end of the 1920s. From then on, DE of metal ores stabilized and remained between 0.3 and 0.4 Gt/yr.

Prior to World War II (WWII), trade flows were small compared to DE; after WWII trade began to soar. In 2005, imports were 15% the size of DE and exports were 5% the size of DE. The United States was a net exporter of materials for the first 75 years of the observed time period. Only after WWII did imports outgrow exports and the physical trade balance turn positive, with an increase in net imports to almost 0.8 Gt/yr, or 9% of DMC. Net trade is dominated by fossil energy carriers, which accounted for more than 90% of net imports in 2005. Net imports amounted to 29% of DMC in this material group. On closer examination, the United States had been self-sufficient with respect to fossil fuels for most of the observed period (until 1957), and even exported large amounts of coal and oil. Exports peaked at 70 megatons (Mt)^7^ in 1947. After WWII, imports grew rapidly, and in 1957 the United States became a net importer of fossil fuels. Since then, net imports of oil and natural gas have been growing and only slumped for a few years when oil prices grew sharply in the 1970s. The United States remains a major exporter of biomass. Net exports of biomass grew throughout the nineteenth century and reached a first peak of 13 Mt around 1900, then began to decline. For several decades, imports of biomass exceeded exports; after 1960, exports surged and net exports increased rapidly to a peak of 135 Mt in 1981. During the most recent years, net biomass exports have stabilized at around 80 Mt/yr. Physical trade of ores and nonmetallic minerals was roughly balanced for most of the observed period. Net imports of ores began to surge after WWII, and the same for nonmetallic minerals in the 1970s.

Throughout the observed period, net trade was small compared to domestic extraction. Consequently the difference between DE and DMC in terms of size, composition, and development over time is small with the exception of fossil fuels, in which case DMC became considerably larger than DE (figure 1). The share of fossil fuels in DMC in 2005 was 33% (compared to 23% in DE). Total DMC increased steadily over the whole period and rose from 0.4 Gt in 1870 to 8.1 Gt in 2005. Similar to DE, all periods of economic disruptions or recession appeared as a temporary decline in DMC.

## Sociometabolic Transitions

This section discusses the U.S. transition from an agrarian to an industrial sociometabolic regime (Krausmann et al. 2008b; Haberl et al. 2011). In line with observations from other case studies (Krausmann et al. 2009, 2011), we distinguished phases that differ with respect to the composition and growth rates of material and energy use and the level of per capita consumption (see table 1).

**Table 1:** Average annual U.S. growth rates of population, gross domestic product (GDP), and resource use during different periods of the metabolic transition and factor growth between 1870 and 2005 levels

	*1870–1929*	*1932–1973*	*1984–2005*	*Factor 2005/1870*
Population	1.9%	1.3%	1.0%	7.5
GDP, in intl. $	3.7%	4.4%	3.1%	91.6
DMC, in kg	2.8%	3.3%	1.5%	19.0
TPES, in J	4.1%	3.3%	0.8%	36.5
Income (GDP per capita), in intl. $/cap/yr	1.7%	3.0%	2.0%	12.2
DMC, in t/cap/yr	0.8%	2.0%	0.4%	2.5
DMC minerals and fossils, in t/cap/yr	3.3%	3.2%	0.7%	13.4
TPES, in GJ/cap/yr	2.1%	2.0%	−0.3%	4.9

*Note:* 1870–1929: coal phase of the metabolic transition; 1932–1973: oil-based growth; 1984–2005: consolidation of industrial metabolism. We do not show average growth rates for the years after the Great Depression (1929–1932) or the years after the first two oil price shocks (1974–1983), which were characterized by strong fluctuations in material and energy use.

*Sources:*U.S. Bureau of the Census (1975), FAO (2009) (population); Maddison (2008) (GDP); all other data: own calculations based on MFA database. intl. $= international dollars; kg = kilograms; J = joules; DMC = domestic material consumption; TPES = total primary energy supply; intl. $/cap/yr = international dollars per capita per year; t/cap/yr = tonnes per capita per year; GJ/cap/yr = gigajoules per capita per year.

### The Coal Phase of the Metabolic Transition

**Figure 2:**
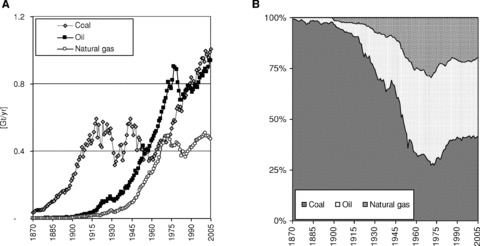
(a) Domestic material consumption (DMC) of fossil energy carriers in the U.S. in gigatons per year (Gt/yr) and (b) U.S. DMC by share of total fossil energy carrier.

We begin to observe the U.S. physical economy in a period of economic and metabolic transition. After the Civil War (1861–1865), the United States moved rapidly from an agrarian to an industrial regime and followed a pathway of coal-based industrialization, much like the United Kingdom several decades earlier (Schandl and Schulz 2002; Krausmann et al. 2008c). In the 59 years from 1870 to the Great Depression, coal extraction and consumption increased 15-fold (figure 2). By 1920, domestic consumption of coal reached a first peak of 5.6 tonnes^8^ per capita per year (t/cap/yr), and then slowly declined. In this period, U.S. per capita coal consumption exceeded that of the United Kingdom, which was around 4 t/cap/yr. A considerable amount of coal was even exported by the United States. In association with coal, iron and steel production surged. It grew from less than 2 kg/cap/yr in 1870 to 408 kg/cap/yr in 1929, when the U.S. share of global production reached 50%. The railroad network expanded from 85,000 to 410,000 kilometers (km),^9^ opening up the resource-rich continent. Settlement and cultivation expanded westward. Between 1870 and 1930, roughly 405,000 square kilometers (km^2^) of fertile prairie land^10^ were plowed up in the Great Plains region (Cunfer 2005). Agricultural production multiplied and the railway delivered midwestern grain and meat to growing domestic urban markets (Cronon 1992). Agricultural output far exceeded domestic demand, and the United States emerged as a major exporter of agricultural products. At the turn of the century, around 15 million tonnes (15 megatonnes, Mt) of crops and animal products were exported; 5.5 Mt of grain were exported to the United Kingdom alone (K. K. Ackerbauministerium 1900). But this heyday of exports did not last. Cropland expansion reached its limits and could not keep up with high population growth and quickly rising demand for animal products. Net exports peaked in 1898, and the United States turned into a net importer of biomass by the 1920s.

During this period, the United States gained its position as a dominant economic world power. In 1901, U.S. GDP per capita^11^ overtook that of the United Kingdom (Maddison 2008) and immigration contributed to rapid population growth. The United States exploited its rich natural resource base and emerged as a major net exporter of natural resources, providing the industrializing economies in Europe with food and fuel (coal, petroleum). The coal phase of the metabolic transition was characterized by a rapid decline in the share of renewable biomass in DMC. Already by 1911 it had fallen below 50%, and it continued to decline despite domestic consumption of biomass more than doubling. DMC, and in particular the consumption of mineral and fossil materials, grew quickly, with rates exceeding that of population (table 1). Physical growth (growth of DMC) even accelerated around 1900. Overall, DMC grew from 11 to 18 t/cap/yr during the coal phase of the metabolic transition.

### Oil and the Emergence of Mass Production and Consumption

**Figure 3:**
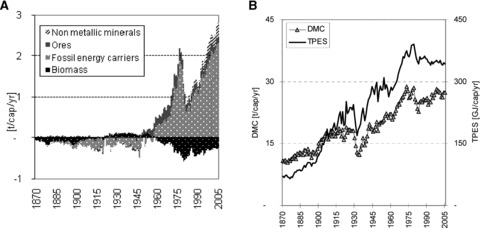
(a) Physical trade balance (PTB) of the U.S. in tonnes per capita per year (t/cap/yr) and (b) U.S. metabolic rates (material use [domestic material consumption (DMC)] and energy use [total primary energy supply (TPES)] per capita per year). PTB is calculated as physical imports minus exports. Negative values designate net exports.

The Wall Street crash of 1929 and the following Great Depression had an enormous impact on the U.S. economy. Alongside GDP, the physical economy also plummeted. Material and energy use slumped by roughly 30% in the years after 1929 (figure 3b). But after only four years the physical economy recovered and material and energy use started to grow at unprecedented rates (see also table 1). The 1929 level of resource use was surpassed around 1940. Recovery of the physical economy was supported by New Deal measures: large construction projects were launched, electrification of peripheral areas was pushed forward, structural adjustments in agriculture were promoted, and the Social Security Act was enacted (Adams 2008). The beginning of WWII, which boosted U.S. industrial capacity and considerably damaged its major industrial competitors, accelerated economic growth, the transition from coal to oil, and the consumption of mineral and fossil materials, though this process had begun before the crisis with the emergence of a new technological cluster consisting of oil, automobiles, the chemical industry, and electricity (Grübler 1998). Combined with a new socioeconomic order, this acceleration formed the basis for a new pattern of material and energy use driven by mass production and mass consumption (Cohen 2003). Oil, and later natural gas from domestic deposits, increasingly supplemented coal in the energy system and overtook coal as the dominant energy carrier in the 1940s. Similar to biomass, however, coal was not replaced by oil and natural gas, but remained an important energy carrier. In the course of WWII the use of coal gained in importance and reached the same level as in 1920. After WWII the use of coal sharply declined for a few years, but began to increase again in 1958, surpassing the peak use of the 1920s in 1979 (figure 2a). The increasing significance of coal in the 1970s was at least partially connected to the oil price shocks and a reverse substitution of coal in some functions for which it had previously been replaced by oil.

The period between the Great Depression and the oil price shocks of the 1970s was a period of rapid physical growth. DMC grew at an average rate of 3.3% per year (table 1). Declining resource prices and quickly rising wealth paired with motorization, suburbanization, and electrification of households were primary drivers of resource use. Material and energy use grew much faster than population: DMC per capita more than doubled, from 13 t/cap/yr in 1932 to 29 t/cap/yr in 1973, and TPES per capita grew from 169 gigajoules^12^ per capita per year (GJ/cap/yr) to 375 GJ/cap/yr in the same period (figure 3b). The use of nonmetallic minerals for construction multiplied sixfold to 12 t/cap/yr, and from 1951 onward nonmetallic minerals had the highest share of total DMC. Major infrastructure projects, such as the development of the interstate highway system beginning in 1950, contributed to the growth of resource use. Construction is a major driver of demand for mineral materials and ores. It has been shown for the United States that a large fraction of steel and copper is used in infrastructure and buildings (Gordon et al. 2006; Müller et al. 2006). The USGS (Sullivan 2006) estimates that the current 73,000 km of interstate highway alone (of a total road network of more than 6.5 million km) corresponds to a stock of 1.5 Gt of natural aggregates, 48 Mt of asphalt, and 6 Mt of steel. The massive diffusion of motor vehicles also contributed to rising resource use: Between 1945 and 1973, the number of registered cars rose from 182 to more than 600 vehicles per 1,000 inhabitants (U.S. Bureau of the Census 1975, RITA 2010), accounting for 7% of all steel stocks in the United States (USGS 2006).

With this quickly growing resource demand, the U.S. economy became a net importing economy. In 1958, the United States turned from a net exporter of fossil energy carriers to a net importer. In 1973, already 20% of all fossil energy carriers and one-quarter of all petroleum and natural gas consumed in the United States were imported. Net imports of ores and metals began to increase in the late 1940s, while imports of nonmetallic minerals increased in the 1970s (figures 1c and 3a). Only biomass exhibited a different trend: After four decades of net imports, the industrialization of agriculture boosted crop production and the United States became a net exporter of crops and agricultural products. The green revolution once again made the United States one of the world's largest exporters of agricultural products, in particular wheat, corn, and soybeans.

This phase of the metabolic transition was characterized by the rapid expansion of physical stocks and fast growth of per capita use of all material groups. The typical pattern of an industrial metabolism emerged in close association with the establishment of the American way of life and its patterns of mass production and consumption.

### Consolidation of the Industrial Metabolism after the Oil Price Shocks?

The oil price shocks in 1973 and 1979 ended three decades of fast and continuous physical growth and contributed to a severe recession in the early 1980s. Oil imports plummeted, and, in 1970, domestic extraction of petroleum also reached its peak and began to decline. Along with energy consumption, growth in all other materials came to a halt. As with the Great Depression, the physical economy recovered after a few years. Oil imports began to rise again. In the aftermath of high oil prices and peak extraction, domestic coal was rediscovered as an energy carrier and gained significance in the energy system (figure 2). Coal extraction and use increased continuously, and in 2005 exceeded previous peak values by 60%; in contrast, domestic extraction of oil was 43% below the 1970 peak. After several ups and downs, DMC began to rise again in 1984, but at considerably lower rates than before (table 1). Material and energy use grew at a rate similar to population, yet by 2005 metabolic rates had not reached a level equivalent to that prior to the first oil price shock. The peak of per capita DMC was reached in 1973 at 29 t/cap/yr, and that of TPES in 1979 at 391 GJ/cap/yr (figure 3b). The overall amount of DMC and TPES, however, continued to grow: by 2005, material and energy use was roughly 30% greater than the 1973 level. Import dependency on fossil fuels reached a new maximum, and in 2005 roughly one-third of all U.S. fossil energy carriers (in terms of mass units) were imported. The oil price shocks had a massive short-term impact, but they also had some lasting impacts on the structure of the economy, the pace of physical growth, and the composition of material and energy use (Ayres 2006).

While in most industrialized countries the growth of material and energy use slowed down considerably after the oil price shocks in the 1970s, it remains an open question if the observed stabilization of metabolic rates should be interpreted as a consolidation of the industrial metabolic pattern. While the industrial regime has reached a certain state of maturity in terms of built infrastructures and productive capacity, the stabilization of material and energy flows per capita may also be a result of a shift toward less material-intensive service industries domestically, and an outsourcing of material-intensive production (cf. Munoz 2009; Peters et al. 2011).^13^

## Material Use and Economic Development

**Figure 4:**
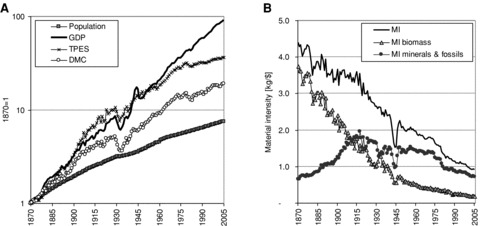
Material use and economic development in the United States: (a) development of population, gross domestic product (GDP), domestic material consumption (DMC), and total primary energy supply (TPES); and (b) material intensity (MI) in kilograms per unit GDP. MI is defined as DMC/GDP. Note the logarithmic scale in Figure 4a. *Sources:*U.S. Bureau of the Census (1975), FAO (2009) (population); Maddison (2008) (GDP); all other data: own calculation based on MFA database.

Figure 4a shows the evolution of material and energy use in comparison with GDP and population growth. During the observed 135-year time period, DMC grew by a factor of 18 and TPES by a factor of 37. Population increased only sevenfold, while the economy increased by almost two orders of magnitude, considerably faster than resource use. As a consequence, the amount of materials (and energy) used per unit of GDP (material intensity) declined substantially. The U.S. economy thus exhibits a pattern of relative dematerialization (or relative decoupling) (UNEP 2011).

Figure 4a shows that energy consumption had been rising very much in line with GDP for almost a century, and decoupling started only in the 1970s. Material use, in contrast, grew much slower than the economy during the whole twentieth century. This resulted in a significant reduction in material intensity (figure 4b), which improved by a factor of four: In the 1870s more than 4 kg of materials were used per unit of GDP; at the beginning of the twenty-first century, this ratio was down to less than 1 kg per unit GDP. But figure 4b also illustrates that material intensity did not decline equally for all material groups. While biomass intensity declined over the entire period, mineral and fossil materials were linked more tightly to economic development. Material intensity for this group, just like energy intensity, even increased during the first 45 years, and only after that started to decline. Similar trends have been observed for many key materials of industrialization. Most of the improvements in material intensity of key materials and energy carriers have actually only occurred since the 1970s. This further justifies conceiving of the period after the oil shocks of the 1970s as a distinct phase in the industrial metabolism of the United States.

During the last 135 years, resource use only declined during periods of recession or severe economic crisis. Absolute dematerialization has not been achieved in spite of massive efficiency gains in many industrial processes and in the end uses of material and energy (Ayres et al. 2004). All resource savings have been offset by rising demand induced by declining prices and growing income (Sullivan et al. 2000; Ayres et al. 2003). Also, a shift from products toward services in the economy in the last decades has only contributed to relative dematerialization, while overall material and energy use continued to grow. At the turn of the twenty-first century, each additional dollar of GDP still required roughly 0.2 kg of renewable biomass, 0.7 kg of mineral and fossil materials, and 11 megajoules (MJ)^14^ of primary energy.

## The Physical Economy of the United States in International Comparison

**Figure 5:**
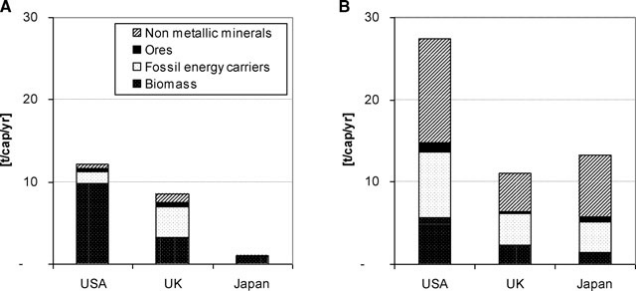
(a) Domestic material consumption (DMC) in tonnes per capita per year (t/cap/yr) in the United States, United Kingdom, and Japan for 1880; and (b) for 2005. *Sources:* Based on Schandl and Schulz (2002), Eurostat (2007), and Krausmann et al. (2011).

The United States has not only dominated the global economy during the twentieth century, but it has also had a considerable impact on global resource use: According to our calculations, the contribution of the U.S. economy to global material use (Krausmann et al. 2009) expanded from 16% in 1900 to 22% in the late 1950s. In the last 50 years the contribution of the United States to global material use has slowly declined, but remained around 15% in 2005. The U.S. share of global TPES is even larger: It peaked in 1945 when the United States used half of the global primary energy supply, and it accounted for 21% in 2005. The United States, with 5% of the global population, consumes a disproportionately large share of global resources. In terms of per capita DMC, the United States today ranks among the global top 10, surpassed only by extractive economies such as Australia, Chile, and Canada (Krausmann et al. 2008b). Per capita DMC of the United States is 27 t/yr, and higher than that of most industrialized, high-income countries. It is, for example, roughly twice the size of the average Japanese or UK DMC (figure 5b); differences with respect to energy use are even larger. Figure 5 shows that this is not a recent phenomenon, but that the United States had a high rate of materials use even at the very beginning of our time series, far exceeding Japanese or UK DMC in 1880.

What are the underlying factors for this high level of resource use? The United States is sparsely populated compared to Japan and most European countries, but rich in natural resources. This roughly indicates a high endowment of natural resources in relation to population. Relative abundance of natural resources is at least one factor that has contributed to a high level of per capita material and energy use from the very beginning. In 1870, population density was only 4 persons/km^2^ and fertile land was abundant. This facilitated a very high rate of U.S. biomass extraction and use of almost 10 t/cap/yr in 1880, much higher than in the United Kingdom (3.2 t/cap/yr) or Japan (0.9 t/cap/yr). The high number of livestock per capita (780 cattle per 1,000 inhabitants in the United States compared to 290 in the United Kingdom or 30 in Japan) was the single most important cause for the high DMC of biomass. But generous use of abundant fuel wood and timber in households as well as industry and the high rate of exports of agricultural and forestry products also had an influence. Large livestock numbers and high per capita meat consumption (table 2) continue to contribute today to a much higher rate of U.S biomass use compared with the other two countries (figure 5b).

**Table 2:** Drivers of resource use in the United States, the United Kingdom, and Japan. Data for 2005, except when otherwise noted

		*USA*	*UK*	*Japan*
Population density	cap/km^2^	32	249	351
Income (GDP/cap) (const. 2005 $)	$/cap/yr	37,702	27,754	38,972
Household final consumption expenditures	$/cap/yr	26,757	18,626	21,817
Livestock	cattle/1,000 inh.	315	178	35
Meat consumption	kg/cap/yr	124	87	47
Motorization	MV/1,000 inh.	820	478	562
Pump price of gasoline (2000)	US$/L	0.5	1.2	1.1
Passenger road transport	1,000 pkm/cap/yr	26	12	7
Electricity consumption	MWh/cap/yr	14.6	6.8	8.7
Residential electricity consumption	MWh/cap/yr	4.5	1.9	2.6

*Note: Sources:*World Bank Group (2010) (road transport, income, household final expenditure, gasoline price), IEA (2007) (electricity), FAO (2009) (cattle, meat consumption), Mitchell (2003) and RITA (2010) (motorization).

cap/km^2^= individuals per square kilometer; GDP/cap = gross domestic product per capita; const. 2005 $= constant 2005 dollars; $/cap/yr = dollars per capita per year; cattle/1,000 inh. = cattle per 1,000 inhabitants; kg/cap/yr = kilograms per capita per year; MV/1000 inh. = motor vehicless per 1,000 inhabitants; US$/L = U.S. dollars per liter; pkm/cap/yr = passenger kilometers per year; MWh/cap/yr = megawatt hours per capita per year.

In the twentieth century, increasingly large-scale extraction of coal, oil, and mineral resources along with the growing significance of large heavy industries contributed to a high level of DMC. The United States developed a typical metabolic profile of an extractive and export-oriented economy, characteristic today among other new world countries like Canada and Australia (Krausmann et al. 2008b; Schandl and Turner 2009). Figure 5b shows that per capita consumption of each of the four main material groups in the United States in 2005 was much higher than in the United Kingdom or Japan; this was the case during most of the twentieth century. Low and declining prices for energy and materials and high economic growth facilitated resource-intensive industries and consumption patterns and provided little incentive for reduction (Ayres et al. 2004; Sullivan et al. 2000). Mobility and motorization serve as an example: With more than 800 motor vehicles per 1,000 residents, the United States has one of the world's highest rates of motorization (table 2). In an international comparison of energy use, Schipper (2004) concluded that U.S. citizens drive the greatest distances per year, own the greatest number of cars in relation to GDP, and have the highest fuel intensity in terms of fuel use per kilometer (see also table 2). Mobility patterns not only contribute to a high level of fuel use, but building and maintaining a large vehicle fleet and extensive road network in a sparsely populated country have contributed to the high consumption of metals, construction minerals, and fossil energy carriers in the past and present. Low-density settlement patterns, suburbanization, and energy intensive heating and cooling patterns also contribute their share to the high DMC of the United States (Rickwood et al. 2008). Schipper (2004) found that U.S. houses are larger and household appliances more inefficient than in other countries, contributing to the high level of residential energy and electricity use (table 2).

## Footnotes

1 The territorial system boundary used in this MFA refers to the current territory of the United States of America (USA), including Alaska, which was purchased from Russia in 1867, and Hawaii, which was annexed in 1898.

2 For reasons of accessibility we used the 1975 edition of the Historical Statistics of the United States. The most recent edition was published in 2006. The data we have used are identical to those in more recent versions.

3 http://www.rff.org.

4 One kilogram (kg, SI) ≍ 2.204 pounds (lb).

5 A comparison of the results of our estimate of U.S. material extraction and use with results from previous studies is presented in the supporting information on the Web.

6 One gigaton = 10^9^ tonnes (t) = 10^12^ kilograms (kg, SI) ≍ 1.102 × 10^9^ short tons).

7 One megatonne (Mt) = 10^6^ tonnes (t) = one teragram (Tg, SI) ≍ 1.102 × 10^6^ short tons).

8 One metric ton (t) = 10^3^ kilograms (kg, SI) ≍ 1.102 short tons.

9 One kilometer (km, SI) ≍ 0.621 miles (mi); personal calculation based on U.S. Bureau of the Census (1975).

10 One square kilometer (km^2^, SI) = 100 hectares (ha) ≍ 0.386 square miles (sq. mi.) ≍ 247 acres.

11 GDP in international Geary–Khamis dollars.

12 One gigajoule (GJ) = 10^9^ joules (J, SI) ≍ 2.39 × 10^5^ kilocalories (kcal) ≍ 9.48 × 10^5^ British thermal units (BTU).

13 Between 1987 and 2002, the share of foreign-sourced goods in total manufacturing inputs increased from 12.4% to 22.1% (Burke et al. 2004).

14 One megajoule (MJ) = 10^6^ joules (J, SI) ≍ 239 kilocalories (kcal) ≍ 948 British thermal units (BTU).

## Conclusions

The development of the U.S. physical economy during the last century shows the characteristic pattern of a sociometabolic transition: massive growth of both extraction and trade of materials, a shift from the dominance of renewable biomass toward fossil and mineral materials, and a multiplication of metabolic rates. As for other cases, it is possible to distinguish different phases of growth and stabilization, or even slight declines in metabolic rates, since the 1970s. But unlike most other industrial countries, total DMC did not stabilize, but continued to grow. Our results corroborate the findings by Ayres and colleagues (2004) that the United States is not dematerializing to any degree that has environmental significance. Our material flow analysis indicates that population growth obviously plays an important role in this case. Each additional inhabitant with resource-intensive consumption patterns adds to overall material use. But if we acknowledge that high economy-wide flows are also a result of generally material-intensive structures (infrastructure, industry), then population growth may also have another effect: In this case, population growth means that high overall flows are divided by a larger number of people, thus lowering per capita DMC.

We have shown that the U.S. economy is contributing a disproportionately large share to global resource use. Per capita rates of material consumption in the United States are higher than in other industrial countries. This is a result of different underlying factors and can be attributed to a material-intensive lifestyle and consumption patterns of the American way of life, but also to material-intensive infrastructures, settlement and mobility patterns that have evolved over the course of industrialization as a result of biogeographic factors, abundant resources, and declining raw material prices (Rogich et al. 2008). In that sense, our results indicate that high (or low) metabolic rates are not only a result of consumer choices and lifestyles, but of historically rooted patterns of agricultural and industrial production, infrastructure, and settlement patterns. The U.S. example also illustrates that decisions on the spatial organization of the economy and infrastructure have a long-lasting effect on resource use and the resource intensity of housing and mobility patterns. The exact interplay of the underlying factors that determine material use is far from fully understood and requires further research. A better understanding of these factors, their interplay, and how they materialize in historical legacies that shape future resource use patterns are essential to answering the question of where measures and policies in support of dematerialization might best be applied.

## About the Authors

**Sylvia Gierlinger** is a researcher and **Fridolin Krausmann** is a professor at the Institute of Social Ecology at Klagenfurt University in Vienna, Austria.

## Supporting information

**Supporting Information S1:** This supporting information provides a comparison of the authors' estimate of U.S. material extraction and use with results from previous studies.
