# Supplementary material for: The Physical Economy of the United States of America
Source: J Ind Ecol. 2011 Dec 1;16(3):365–77. doi: 10.1111/j.1530-9290.2011.00404.x (PMC3886303; doi:10.1111/j.1530-9290.2011.00404.x)
Supplement: Supplementary file 1 [file jiec0016-0365-SD1.zip › 3740sg2r - Replacement suppinfo v2.pdf]

SUPPORTING INFORMATION FOR:

Gierlinger, S. and F. Krausmann. 2012. The Physical Economy of the United States of America: Extraction, Trade and Consumption of Materials from 1870 to 2005. *Journal of Industrial Ecology.*

Summary

This supporting information provides a comparison of the authors' estimate of U.S. material extraction and use with the results from previous studies.

1. Trade flows

Due to limited availability of trade data, our series on physical imports and exports includes raw materials and semi-manufactured products only, while trade of many manufactured products, such as vehicles, machinery, chemicals, plastic products, or furniture was omitted. In order to get a quantitative understanding of the significance of the omission of these flows, we crosschecked our results with data from the UN's comtrade data base (United Nations Statistical Division, 2008). Comtrade data are available for the period 1962 to the present, but this database also reports fragmentary data for physical trade for most years. More or less complete coverage of mass flows of imports and exports exists only for the years 1978, 1985-1988 and 2005. A comparison of trade estimates that includes manufactured products from the comtrade database and the trade flows covered in our data series for the years 1978 and 2005 is presented in Figure S-1. It shows that manufactured products not considered in our account amount to 10-20% of total imports and 4-11% of total exports. Underestimation is highest for imports and exports of metal products (vehicles, machinery), petrochemical products (organic chemicals, plastics) and so called "products" (products which cannot be assigned to a specific material group). However, in terms of net trade (PTB), the underestimation is much lower and amounted to -1% in 1978 and 6% in 2005. Although the omission of manufactured products causes considerable distortions for some material groups, the effect on aggregate flows and indicators of material use is rather insignificant. The impact on the size and time trend of DMC or PTB of the four main material groups is small.

Figure S-1: Comparison of trade data from different sources for the years 1978 and 2005. Estimates of trade flows presented in this article ("this estimate") are compared to an estimate that includes data on manufactured products from UN's comtrade database ("comtrade"). The classification system used to allocate trade items to material groups follows Eurostat 2009: Manufactured products are attributed to material groups according to their main raw material component; in cases where this was not possible, they were assigned to the group "products". Physical trade balance (S-1e and f) is defined as imports minus exports. Negative values indicate net exports. Sources: "Comtrade" combines raw material trade as reported by FAO 2009 and IEA 2007 with trade of manufactured products as reported in comtrade (United Nations Statistical Division, 2008).

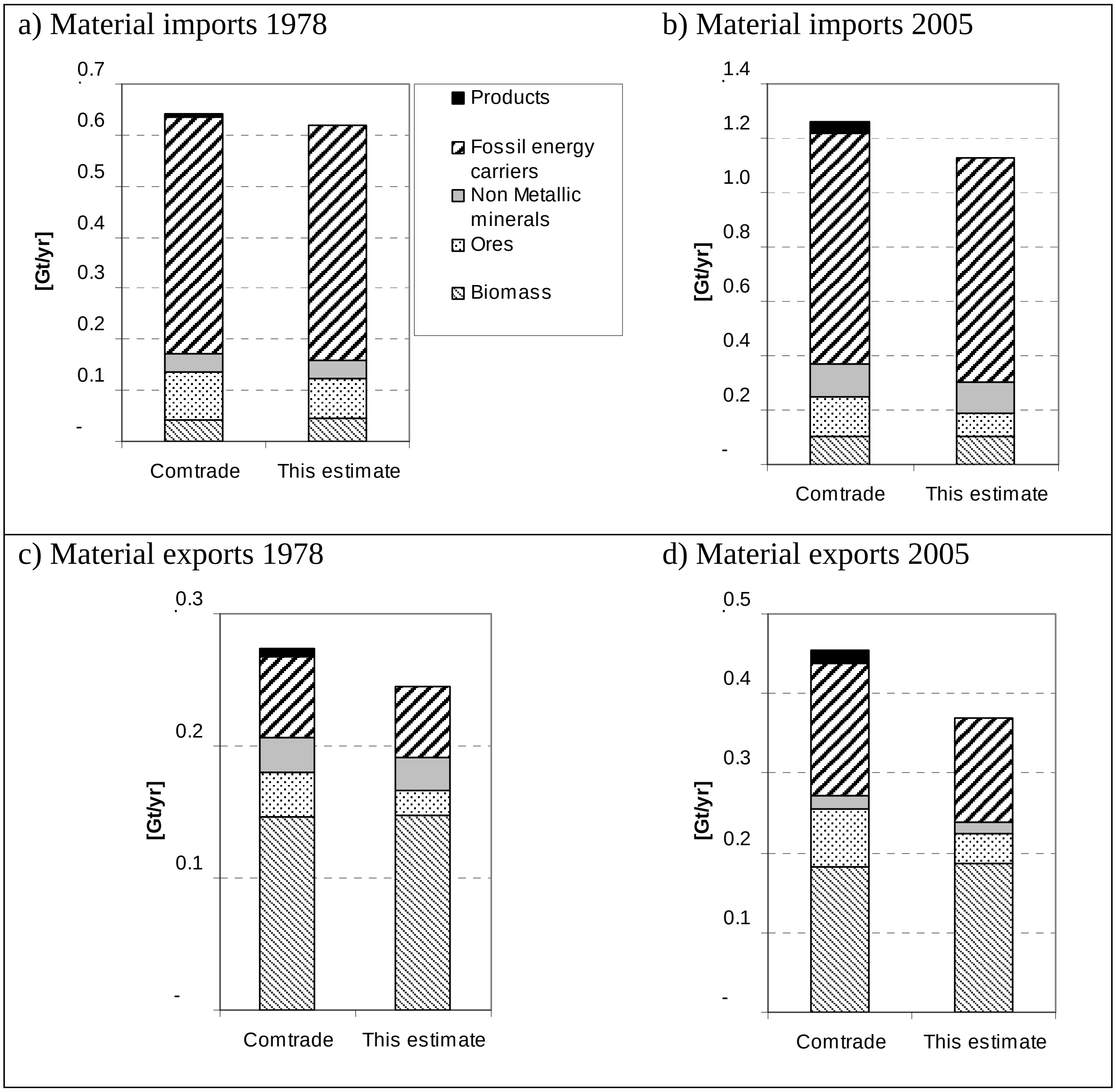

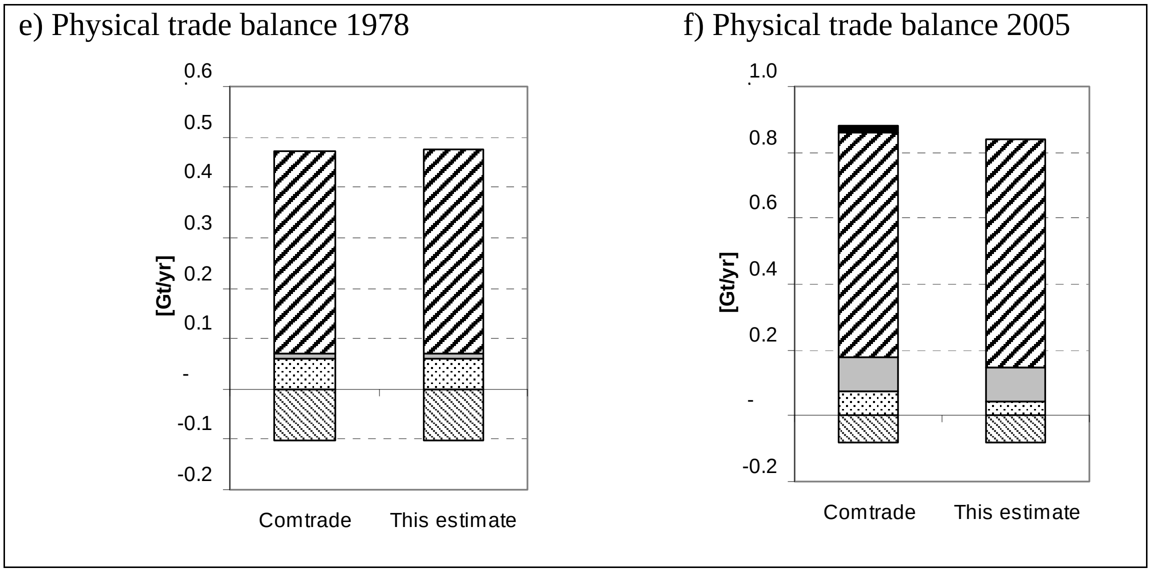

2. Extraction of sand, gravel and crushed stone

Although quantitative information on the use of natural aggregates (sand, gravel and crushed stone) is available from statistical sources, we applied an estimation procedure to quantify the extraction of this large fraction of non metallic minerals. On the one hand, data on the extraction of natural aggregates reported in HSUS, 1975 are not consistent with the much lower values provided in Kelly and Matos, 2008. Results presented by Matos, 2009 also indicate significantly higher values. On the other hand, we wanted to ensure the highest possible degree of comparability with other long term MFA studies. We applied an estimation procedure based on the guidelines of material flow accounting of Eurostat, 2009. This procedure is widely used and has been adapted for the application in other world regions (e.g. Schandl and West, 2010, Steinberger et al., 2010) and long term historical studies (Krausmann et al., 2009; Krausmann et al., 2011). In contrast to other available estimation procedures, which use monetary information to quantify the use of so called construction minerals, the estimate applied in this study is based on purely biophysical data. It uses data on the production and use of cement, concrete, and asphalt to extrapolate the demand for natural aggregates. We applied standard coefficients on the ratio of sand and gravel to cement in concrete and sand and gravel to bitumen in asphalt to extrapolate natural aggregates. Based on Krausmann and colleagues (2009) we assumed a ratio of sand and gravel to cement in concrete of 6.1 and of gravel to bitumen in asphalt of 20. Furthermore we assumed that 1.15 tonnes of limestone are required to produce one tonne of cement. In order to account for sand and gravel use as filling material in road construction and in the considerable network of unpaved roads, we increased the asphalt estimate by 50% (cf. Steinberger et al., 2010). Data on the production and consumption of cement and bitumen were taken from HSUS 1975, Kelly and Matos 2008, IEA 2007 and Abraham, 1945.

The results of our estimate, which we regard as conservative, are significantly higher than the values for sand and gravel used for construction reported by Kelly and Matos 2008

and match remarkably well with the more comprehensive figures presented in the USGS estimate of minerals use (Matos 2009). Figure S-2 shows that our estimate of non-metallic minerals is roughly 20% above the values reported in Matos (2009), and that trends over time are highly consistent in both estimates.

Figure S-2: Use of non-metallic minerals in the USA, 1900–2005: Comparison of data provided in Matos (2009) and this estimate.

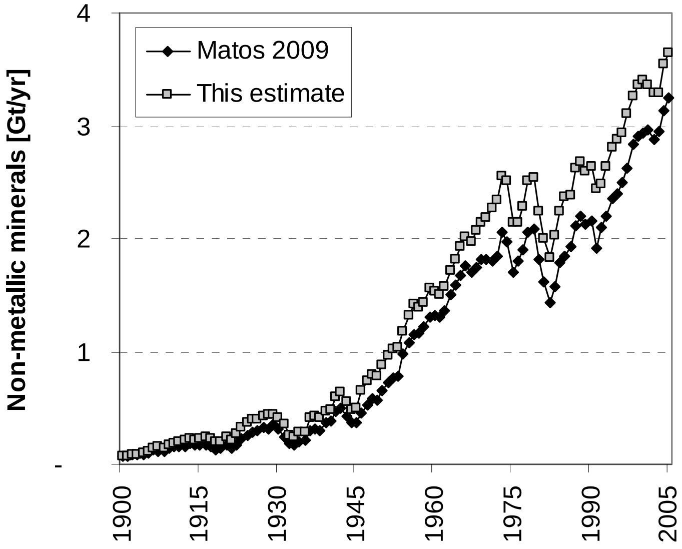

3. Primary energy sources

Various sources covering data on primary energy supply exist for the United States (e.g. BP 2009; IEA 2007; UNSD 2011). Most cover a period of a few decades only. While all provide very similar trends, deviations in the amount of energy flows of several percent occur due to minor differences in system boundaries, applied conversion coefficients, and primary data used. In order to avoid statistical breaks in the 135 year time span covered in our study, we calculated a time series of primary energy supply based on the economy wide material flow data from our database. We used average gross calorific values from the literature (Haberl 2001; Haberl et al. 2006) to convert domestic consumption of fuelwood, coal, petroleum, and natural gas from mass into energy units (Joules). We used data on electricity production to extrapolate primary energy inputs from hydropower, nuclear heat, and geothermal sources based on information on conversion efficiency from the literature (Ayres et al. 2003). The resulting TPES series is not identical to other sources but matches well and shows very similar trends. Figure S-3 shows that the overall trend in the development of primary energy supply for the United States from 1960–2005 from our calculation is similar to that reported for TPES in the IEA database, but that the level of our series is between 6 and 13% higher. These differences can be attributed to the use of gross instead of net calorific values, differences in the way electricity from hydro- and nuclear power is accounted for, and the primary data used, particularly for fuel wood.

Figure S-3: Development of primary energy supply in the U.S. from 1870 to 2005. Comparison of data derived from IEA 2007 and this estimate.

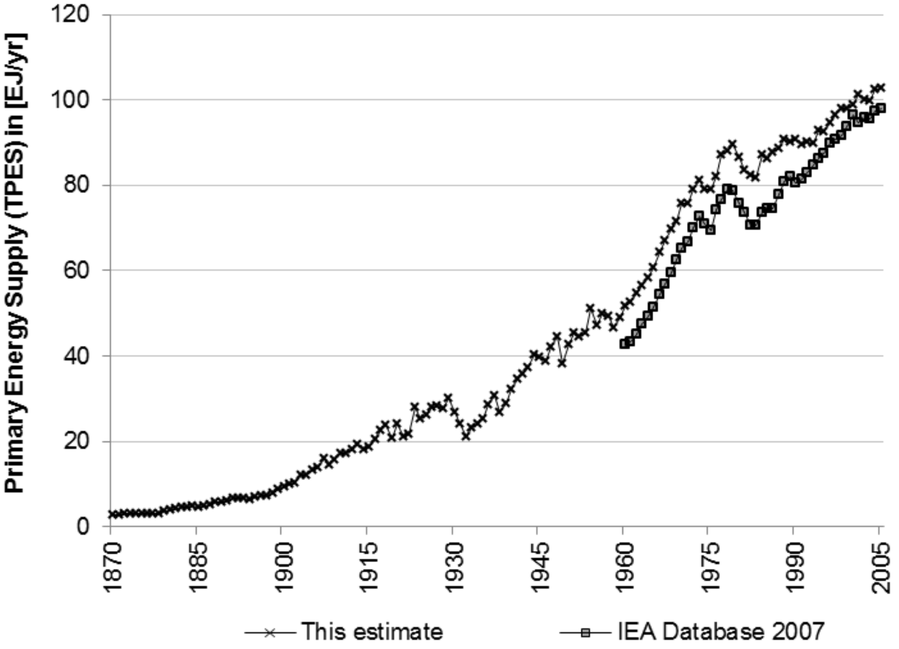

4. Comparison with previous material flow accounts for the USA

As has been outlined in the introduction of this article, several previous studies have explored historical trends of energy and material use in the United States. An initial database compiled by Matos and Wagner (1998) focused on raw materials for industrial use and presented yearly data for the period 1900–1995. It was later updated and currently includes 2006 as the most recent year (Matos, 2009, see also Rogich and Matos, 2002); the data are available for download from the United States Geological Survey webpage. Although covering important parts of social metabolism, these data are not fully consistent with agreed upon MFA system boundaries and accounting principles (Fischer-Kowalski et al., 2011, Eurostat 2009): The estimate focuses on materials used in industrial processes and accounts for materials at the input-to-manufacturing stage, but not materials extracted from nature. Furthermore, it omits the largest part of all biomass fossil energy carriers and only considers biomass used as raw material for industrial processes and fossil energy carriers used as feedstock in the chemical industry. In contrast, recycled materials are, in deviation from MFA standards, accounted for as inputs. Another drawback of this dataset is that it focuses only on apparent consumption and does not distinguish between extraction, imports, or exports. Figure S-4 shows a comparison of the development of aggregate material use in the United States according to Matos (2009) and results of this study. While the overall trend in material use is remarkably similar in both datasets, the omission of biomass and fossil energy carriers results in much lower values of aggregate material use in the Matos (2009) series. Matos (2009) accounts for 12% of all materials in 1900; this share slowly increased up to 40% in the 1960s and remained at that level since.

Figure S-4 Development of aggregate material use in the US from 1900–2005. Comparison of data provided in Matos (2009) and this estimate.

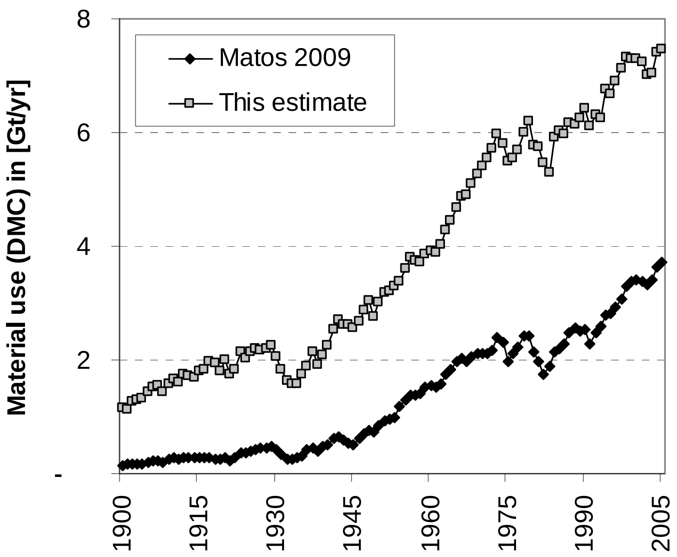

The United States was also one of the case studies in the seminal comparative MFA study initiated by the World Resources Institute (Adriaanse et al., 1997). From this study, detailed MFA data exist, but only for the period 1975–1993; the material flow account was later updated to the year 2000 (Rogich et al., 2008). Figure S-5 compares our estimates of material use with these data. Although the system boundaries and accounting procedures applied in Rogich and colleagues (2008) are similar to those suggested by Eurostat (2009), which have also been used in this study, some important differences remain, in particular because Rogich and colleagues (2008) apply a more restrictive definition of what is accounted for as used extraction and what as hidden flow: They do not account for gross ores or crop residues as used extraction. Also large flows like biomass grazed by livestock are omitted and instead meat and milk are accounted for as inputs. Figure S-5 shows that the overall development of material use is remarkably similar in both material flow accounts, but that Rogich and colleagues (2008) arrive at a roughly 25% lower value for total DMC than we do. This is due to the above mentioned differences in system boundaries and in particular to the less inclusive definition of used extraction applied by Rogich and colleagues (2008). Accordingly, the differences are largest for biomass (crop residues and grazed biomass) and minerals (mine overburden).

Figure S-5: Comparison of DMC according to Rogich et al. 2008 this study. S5a compares aggregate DMC; figure 5b presents data on DMC of biomass, fossil energy carriers and minerals according to Rogich et al. 2008 as percentage of the estimate presented in this study.

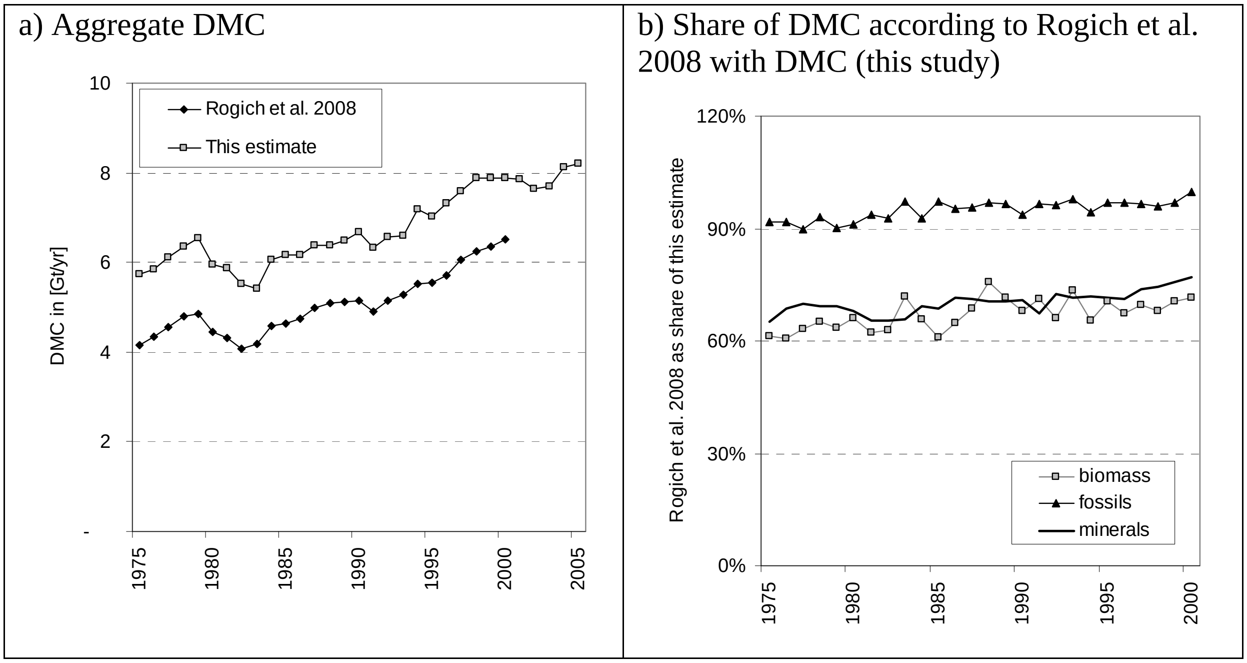

References

Abraham, H., 1945. Asphalts and allied substances. Their occurrence, modes of production, use in the arts and methods of testing. *Volume One: Raw materials and manufactured products*. New York: D. van Nostrand.

Adriaanse, A., S. Bringezu, A. Hammond, Y. Moriguchi, E. Rodenburg, D. Rogich, H. Schütz. 1997. *Resource Flows: The Material Basis of Industrial Economies.* Washington DC: World Resources Institute.

Ayres, R.U., B. Warr, L. W. Ayres. 2003. Exergy, power and work in the US Economy, 1900-1998. *Energy* 28(3): 219-273.

BP. 2009. *Statistical Review of World Energy 2009.* London, http://www.bp.com/statisticalreview. Accessed 16 June 2011.

Eurostat. 2009. Economy wide Material Flow Accounts. Compilation Guidelines for reporting to the 2009 Eurostat questionnaire (Version 01 - June 2009). Luxembourg, European Statistical Office.

FAO. 2009. FAOSTAT. http://faostat.fao.org/site/573/default.aspx#ancor. Rome, Food and Agriculture Organization (FAO).

Fischer-Kowalski, M., F. Krausmann, S. Giljum, S. Lutter, A. Mayer, S. Bringezu, Y. Moriguchi, H. Schütz, H. Schandl, H. Weisz. 2011. Methodology and indicators of economy wide material flow accounting. State of the art and reliability across sources. *Journal of Industrial Ecology* (accepted for publication 2/2011).

Haberl, H. 2001. The Energetic Metabolism of Societies, Part I: Accounting Concepts. *Journal of Industrial Ecology*. 5(1): 11-33.

Haberl, H., H. Weisz, C. Amann, A. Bondeau, N. Eisenmenger, K.-H. Erb, M. Fischer-Kowalski, F. Krausmann. 2006. The Energetic Metabolism of the European Union and the United States: Decadal Energy Input Time-Series with an Emphasis on Biomass. *Journal of Industrial Ecology* 10(4): 151-171.

HSUS. 1975. Historical Statistics of the United States. Bicentennial Edition. Washington, DC: US Department of Commerce, Bureau of the Census.

IEA. 2007. Energy Statistics of OECD Countries, 2007 Edition, CD-ROM. Paris, International Energy Agency (IEA), Organisation of Economic Co-Operation and Development (OECD).

Kelly, T. D. and G.R. Matos. 2008. Historical Statistics for Mineral and Material Commodities in the United States. Version 3.0. United States Geological Survey.

Krausmann, F., S. Gingrich, N. Eisenmenger, K.-H Erb, H. Haberl, M. Fischer-Kowalski. 2009. Growth in global materials use, GDP and population during the 20th century. *Ecological Economics* 68(10): 2696-2705.

Krausmann, F., S. Gingrich, R. Nourbakhch-Sabet. 2011. The metabolic transition in Japan: A material flow account for the period 1878 to 2005. *Journal of Industrial Ecology* (accepted for publication 2/2011).

Matos, G. 2009. Use of Minerals and Materials in the United States From 1900 Through 2006. U.S. Geological Survey Fact Sheet 2009-3008. http://pubs.usgs.gov/fs/2009/3008/ Reston, VA: USGS.

Matos, G. and L. Wagner. 1998. Consumption of Materials in the United States, 1900-1995. *Annual Review of Energy and the Environment* 23: 107-122.

Rogich, D., A. Cassara, I. Wernick, M. Miranda. 2008. Material Flows in the United States: A Physical Accounting of the U.S. Industrial Economy. WRI Report. Data available at: http://www.wri.org/publication/material-flow-accounts#database.

Rogich, D. G. and G.R. Matos. 2002. Material flow accounts: the USA and the world. In: *A Handbook of Industrial Ecology*, edited by R.U. Ayres and L.W. Ayres. Cheltenham, Northampton, UK: Edward Elgar, pp. 260-277.

Schandl, H. and J. West. 2010. Resource use and resource efficiency in the Asia-Pacific region. *Global Environmental Change* 20(4): 636-647.

Steinberger, J. K., F. Krausmann, and N. Eisenmenger. 2010. Global patterns of material use: a socioeconomic and geophysical analysis. *Ecological Economics* 69(5): 1148-1158.

U.S.Bureau of the Census. 1975. Historical Statistics of the United States, Colonial Times to 1970, Bicentennial Edition, Part II. Washington DC.

United Nations Statistical Division. 2008. UN Commodity Trade Statistics Database (UN Comtrade). http://unstats.un.org/unsd/comtrade/. Accessed 16 June 2011.

UNSD. 2011. United Nations Energy Statistics Database (2008). Electronic database, United Nations Statistics Division.
